# Understanding brain states across spacetime informed by whole-brain modelling

**DOI:** 10.1098/rsta.2021.0247

**Published:** 2022-07-11

**Authors:** Jakub Vohryzek, Joana Cabral, Peter Vuust, Gustavo Deco, Morten L. Kringelbach

**Affiliations:** ^1^ Centre for Eudaimonia and Human Flourishing, Linacre College, University of Oxford, Oxford, UK; ^2^ Center for Music in the Brain, Department of Clinical Medicine, Aarhus University, Aarhus, Denmark; ^3^ Department of Psychiatry, University of Oxford, Oxford, UK; ^4^ Center for Brain and Cognition, Computational Neuroscience Group, Department of Information and Communication Technologies, Universitat Pompeu Fabra, Spain; ^5^ Life and Health Sciences Research Institute, University of Minho, Braga, Portugal; ^6^ Institució Catalana de la Recerca i Estudis Avançats (ICREA), Barcelona, Spain; ^7^ Department of Neuropsychology, Max Planck Institute for Human Cognitive and Brain Sciences, Leipzig, Germany

**Keywords:** complexity, emergence, whole-brain models, connectomics, functional magnetic resonance imaging, spatio-temporal dynamics

## Abstract

In order to survive in a complex environment, the human brain relies on the ability to flexibly adapt ongoing behaviour according to intrinsic and extrinsic signals. This capability has been linked to specific whole-brain activity patterns whose relative stability (order) allows for consistent functioning, supported by sufficient intrinsic instability needed for optimal adaptability. The emergent, spontaneous balance between order and disorder in brain activity over spacetime underpins distinct brain states. For example, depression is characterized by excessively rigid, highly ordered states, while psychedelics can bring about more disordered, sometimes overly flexible states. Recent developments in systems, computational and theoretical neuroscience have started to make inroads into the characterization of such complex dynamics over space and time. Here, we review recent insights drawn from neuroimaging and whole-brain modelling motivating using mechanistic principles from dynamical system theory to study and characterize brain states. We show how different healthy and altered brain states are associated to characteristic spacetime dynamics which in turn may offer insights that in time can inspire new treatments for rebalancing brain states in disease.

This article is part of the theme issue ‘Emergent phenomena in complex physical and socio-technical systems: from cells to societies’.

## 1. Introduction

The brain is a hugely complex system, which is able of (re)producing a plethora of behaviours emerging from spatio-temporal dynamics [1]. Consisting of approximately 100 billion neurones with about 100 trillion synapses between them, this dense network of anatomical and functional interactions has been named the human connectome [2,3]. Recent advances in transcriptomics have further demonstrated the heterogeneity of neuronal anatomy across the human brain [4,5]. The arising dynamics of neuronal interactions on the structural scaffold is further modulated by numerous neurotransmitter systems [6]. It is therefore not surprising that many novel approaches ranging from dynamical system theory [7], information theory [8], turbulence [9] to statistical mechanics [10–13] are needed to understand the brain's complex spatio-temporal dynamics in its entirety [1].

**Figure 1.**
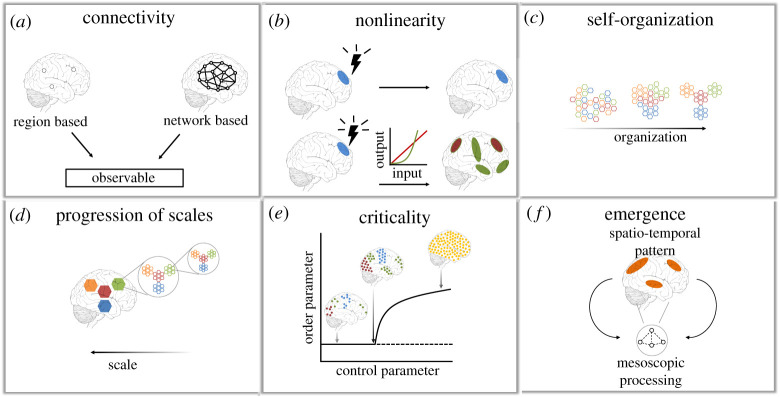
The brain as a complex system. (*a*) A shift in perspective towards considering the brain's function and structure as an integrated network of relationships as opposed to solely localized descriptions of individual regions of interest. (*b*) In many biological systems such as the brain, interactions between stimuli and measurement outputs are mostly nonlinear. (*c*) The spontaneous formation of spatio-temporal patterns from intrinsic brain processes is indicative of self-organization. (*d*) Complex activity patterns are detected across many spatial and temporal scales, from neurones to whole brain, from milliseconds to minutes. (*e*) A system at the edge of instability can have characteristics of critical dynamics. (*f*) The interactions of constituent parts at the mesoscopic scale give rise to brain activity patterns emerging at the macroscopic scale that cannot be merely explained by the individual parts alone (adapted from [1] and [16]). (Online version in colour.)

Indeed, this has meant a paradigm shift away from looking at the brain and its function solely in terms of individual neurones or brain regions to a system-based interaction of a multiplicity of interacting units. One of the fields to emerge from this conceptual leap has been network neuroscience that has focused on describing large-scale structural and functional networks in terms of their properties and relevance for creating complex behaviour [14,15] (figure 1*a*).

In general, the state of a dynamical system can be characterized by the way it responds to external perturbation [17]. For example, in the wakeful brain state, a nonlinear response distributed across the whole cortex is elicited with external transcranial magnetic stimulation (TMS). This is associated to the right balance between differentiation and integration which allows for percolation of the signal throughout the cortex. This contrasts with the deep sleep state, whereby TMS perturbation results in highly localized excitation. Upon further increase in the perturbation strength, the localized response increases but maintains its stereotypical and homogeneous spread unlike the nonlinear response of the wakeful state [18,19] (figure 1*b*). These varying responses of the complex spatio-temporal dynamics recorded with electro-encephalography (EEG) can successfully distinguish between vegetative, minimally conscious or anesthetized states [20].

At the right balance, a complex system can further demonstrate self-organizing properties across space and time from nonlinear interactions of the parts [1,21,22]. Interestingly, this happens in a distributed manner without a centralized control dictating the emerging order [23] (figure 1*c*). In the brain, spatio-temporal organization can be thought of in terms of progression of scales, from the very microscopic (neuronal), mesoscopic (neuronal circuits) to the macroscopic (ensembles of cortical regions). This nested hierarchy has characteristics of scale invariant properties whereby similar features of organization are observed across topology [24], space and time [25] as well as canonical computational motifs [26] (figure 1*d*). Importantly, such organization is hypothesized to happen at the edge of criticality—a dynamical regime where long-range spatial and temporal correlations are made possible [27]. One of the features of systems poised at the edge of criticality is power-law scalings. They have been observed across spatial dimensions—from individual neurones [28] to whole-brain networks derived from functional magnetic resonance imaging (fMRI) [29], as well as across temporal dimensions—both at the fast scale of EEG and magnetoencephalography (MEG) recordings [30,31] and at the slow scale of fMRI data [32]. It is further relevant to appreciate the properties that the system is endowed with close to criticality, as in this regime the dynamic range, capacity and transmission of information are optimized [33,34]. It is in this range that spatio-temporal metastability (a notion of dynamical flexibility) has been hypothesized to be maximal [35–38] (figure 1*e*).

Furthermore, complex systems exhibiting self-organizing properties give rise to emergent phenomena with various examples across nature—flocks of startles, swarms of bees or ant colonies. Such collective behaviour of a system emerges from the interaction of a large number of individual elements, which can only be explained in its entirety by the rules of interaction among parts and not by simply looking at the individual elements alone [39]—the behaviour is said to be computationally irreducible. In the brain, such characteristics are representative of higher order cognition which cannot be simply reduced to the underlying neurophysiology. Specifically, in resting-state brain activity, a condition without any external task, spatially synchronized systems, termed resting-state networks (RSNs), are hypothesized to be emergent from the underlying neuronal activity [26] (figure 1*f*). These emergent properties (examples of weak emergence) result in creating their own rules through which they interact with the environment and having the potential to become the most dominant property determining the activity of the underlying parts [8,40].

## 2. Insights from neuroimaging

Much of the progress in understanding large-scale brain spatio-temporal patterns has come from studying the brain with fMRI, which provides whole-brain coverage at high spatial resolution, at the expense of temporal resolution. A common approach for the analysis of large-scale spatio-temporal activity patterns has been to use a network-based perspective, where static functional connectivity (FC) is estimated as the similarity between the time series of pairs of atlas-based regions [41]. Despite the initial success of shifting the perspective from regional activations to network-based methods, it has remained challenging to tease apart different brain states with sufficient subject specificity using a purely static approach [27,42,43]. Since the brain is a dynamic process that evolves in time, static FC might miss important time-varying characteristics of brain activity [42,44]. Indeed, this has been hinted at by studies focusing on the variability over time of individual functional connections [45].

To this date, many methods have been developed to characterize the fMRI spatial organization varying in time [42,43]. Commonly, various features of time-varying activity are exploited, but largely they converge on quantification of signal variability, spatial substate-based representations and topology of temporal graphs.

**Figure 2.**
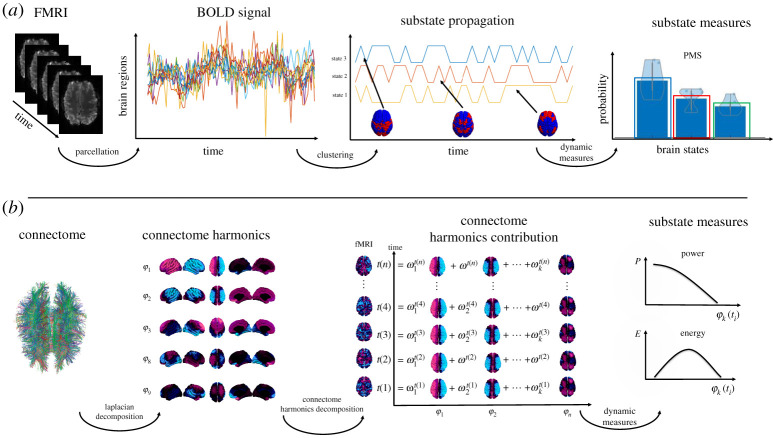
Dynamic approaches to functional MRI. (*a*) Substrate-based representation of fMRI activity. fMRI signals are parcellated into regions; their temporal relationship is quantified and clustered to obtain a set of spatial patterns that dynamically evolve in time. Substrate-based measures allow us to summarize the spatial patterns dynamics. (*b*) Connectome harmonic decomposition (CHD) is an approach that considers spatial patterns expressed from the Laplacian eigenmodes of the structural connectome. The so-called connectome harmonics are then projected onto the time series allowing for analysis of these connectome harmonics in time (adapted from [46]). (Online version in colour.)

While methodological considerations might differ based on a specific approach, they carry a similar outlook on the FC dynamics (FCD) as resulting from the combination of a repertoire of spatio-temporal brain substates. Once such a description is achieved, it is possible to describe spatial substates varying in time in terms of their fractional occupancy (i.e. probability), dwell time (i.e. duration) and transition probability among other summary measures and in turn create a more accurate description of the dynamic processes that the brain engages in (figure 2*a*).

While FCD tries to represent spatio-temporal patterns from brain activity recordings alone, an active area of research has focused on the underlying network of white-matter fibres, derived from diffusion weighted imaging, which enables the emergence of brain activity in different brain states. How structure sculpts function is far from clear; however, it is commonly accepted that brain structure constrains the space on which dynamics emerge. The idea can be put forward in Aristotle's quote ‘the shape of water is determined by its container’. The harmonic modes of diffusion in a structural network can be analytically determined from the eigenvectors of the graph Laplacian [47,48]. Specifically, the connectome harmonics framework has shown that combining a few of the slowest modes of diffusion in the structural connectome (captured from the Laplace eigenvectors with smallest eigenvalue) can accurately describe the known RSNs [47]. Incidentally, these harmonic modes can be approached as building blocks of brain activity to represent complex spatio-temporal patterns of brain activity in mental disorders, as well as in different states of consciousness [49] (figure 2*b*).

## 3. Insights from whole-brain modelling

While FCD offers important insights about the spatio-temporal brain activity, it is crucial to move beyond merely comparative approaches of empirical results to understand how complex dynamics emerges from structural brain topology. Therefore, it is pertinent to construct computational models that will enable us to approximate emerging brain dynamics from the structural connectome through simulation and as such provide fundamental observations about the structural, functional and dynamical properties of spatio-temporal brain activity in different brain states [50,51].

**Figure 3.**
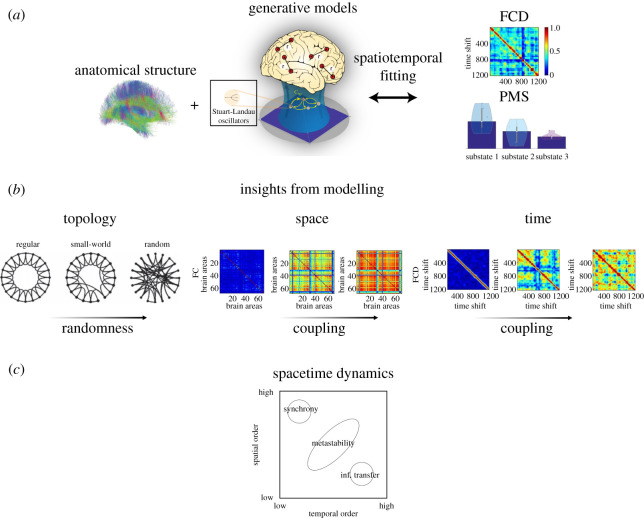
Insights from whole-brain modelling. (*a*) Whole-brain models describe spatio-temporal dynamics in terms of stochastic nonlinear dynamics embedded in each region, which interact with other regions through the anatomical structure represented by the connectome. An important step in the description of such models is validation with empirical FC features. (*b*) Spatial and temporal organization of brain dynamics is preserved in models with structural connectomes exhibiting small-world properties [58] weakly coupled interactions between regions of interest and local dynamics poised at the edge of instability [59]. (*c*) The metastable regime of rich spontaneous brain dynamics can be perceived in-between the extreme cases of the spatial and temporal order continuum (adapted from [60]). (Online version in colour.)

Whole-brain computational models describe neural activity of interacting brain regions as a set of coupled differential equations representing the desired neurophysiology or dynamic profile. To reinforce the biological plausibility of the model, a structural connectome is used to reflect the strength of connections between individual brain regions. The choice of the brain regional model often depends on striking a delicate balance between model complexity and realism. In particular cases, emergent brain dynamics can be addressed through mean-field approximations of neural mass activity or phenomenological models of coupled oscillators [52,53]. Several different scenarios have been proposed, with conductance-based and excitatory-inhibitory neurone-based models describing aspects of brain physiology [54,55] to phenomenological models depicting synchronization mechanisms, such as Kuramoto and Hopf models [56,57] (figure 3*a*).

One of the important aspects of the emerging richness of spatio-temporal activity is its underlying structural connectivity. It has been shown that the optimal fit between empirical and simulated data emerges when modularity and efficiency are balanced [61], which is directly linked to the topological properties of the structural connectome [62]. Importantly, when such topology is disrupted through lesioning of the underlying connectivity, many important properties of the emerging dynamics are lost [63,64]. Moreover, when simulating dynamics across a range of network architectures, from the regular lattice topology to random network organization, the optimal working point emerges in the intermediate small-world regime demonstrating both high modularity and high efficiency [61].

Another important aspect driving the emergence of spatio-temporal features is the coupling strength between neuronal populations. At the optimal weakly coupled point, neuronal populations have the ability to influence one another resulting in collective activity patterns that approximate RSNs [55] and static FC [52]. Instead, if the coupling is too strong, complete synchronization of the neuronal populations results in the loss of functional specificity. On the other hand, at very little coupling, the activity is governed by the local neuronal populations rendering the emergent spatial patterns structureless [57]. Furthermore, the delays arising from the transmission of signals between neuronal populations have also been shown to be relevant [55,56,65], although they seem to affect the temporal and spectral properties of RSNs rather than their integrity [7].

FC evolves over time, and therefore, it is pertinent to consider the mechanisms giving rise to such spatio-temporal fluctuations. In other words, applied whole-brain models should further illuminate FCD features beyond static FC or the emergence of RSNs. Recently, it has been demonstrated that at an optimal level of coupling between brain regions, structured noise alone (combined with the SC) can explain the static FC, but not the non-stationary dynamics [66,67]. This begs the question: ‘What are the additional principles giving rise to such spatio-temporal dynamics?’ One possibility is to attribute it to stochastic nonlinear dynamics embedded in each region. Accordingly, the spatio-temporal dynamics emerges from noise-induced fluctuations of regional nonlinear models poised at the edge of instability [68]. As such, the model becomes endowed with characteristics of critical systems such as an increase in the repertoire of possible brain substates and long-range temporal correlations [16,69]. Indeed, such characteristics have been demonstrated to emerge from coupled oscillatory units with fluctuating amplitude, represented by a supercritical Hopf bifurcation, where the whole-brain network model was able to recapitulate spatio-temporal measures including FCD [57] and probabilistic metastable substates [70].

## 4. Spacetime of psychedelic and depressive brain states

With the development of whole-brain non-invasive neuroimaging techniques, it has been recognized that different brain states are made up of waning and waxing of evolving spatio-temporal patterns [71]. While optimal functioning of the human brain can be recognized in the resting-state condition of ordinary waking state, it becomes altered in other brain states such as the psychedelic-induced state or clinically determined depressive state. Importantly, the hypothesis is that the relationship between the psychedelic-induced and depressive state can be approached from a theoretical perspective combining insights from spatio-temporal analysis of neuroimaging data with whole-brain network models.

## 5. Theoretical descriptions

In the entropic brain hypothesis, it is posited that the level of entropy of complex brain activity (understood broadly in terms of neural signal diversity) indexes the richness of informational content of brain states with upper and lower boundaries marking the cessation of ordinary waking state. Positioned in a zone of instability, the ordinary waking state is observed with a sufficient stability and flexibility. When entering the psychedelic-induced state, entropy is enhanced resulting in more susceptible and malleable brain dynamics. The spontaneous brain dynamics is believed to move closer to criticality with a broadening of the repertoire of possible substates that the brain engages in [49]. Conversely, in the depressive state, such dynamics is characteristically inflexible with ruminative and self-critical periods of thinking resulting in diminished entropy. In this respect, the ability to flexibly engage in divergent thinking becomes impaired. One possibility is that the brain dynamics become less metastable, with a given substate, for example the default mode network (DMN) and fronto-parietal network (FPN), coming to control most of cognition [72,73].

A further description of psychedelic-induced and depressive brain states in terms of large-scale functional networks and spatio-temporal dynamics is the RElaxed Beliefs Under pSychedelics (REBUS) model. In this perspective, psychedelics are acting to relax precision of high-level priors or beliefs and thus making them more sensitive to the bottom-up information inputs, predominantly through the limbic system, that would otherwise be omitted, and potentially revising, and cultivating aberrant priors. These high-level priors are encoded in spontaneous activity of neuronal hierarchies, especially in high-level associative regions as well as the DMN, acting as compressive or summary models that constrain the content of the levels below. By relaxing/decompressing these priors, it is possible for the unheard or suppressed information to travel freely through the neuronal hierarchies and be noticed in higher levels [74]. The REBUS principle implies the anarchic brain whereby the intrinsic hierarchy of information processing is disrupted mainly at the higher levels, as represented for example by the FPN and DMN. This results in bottom–up information flows being put on the same footing with higher levels of the hierarchy. In this sense, there is no longer any ‘central control’, as implied by the term anarchic, resulting in the loss of the functional hierarchy, enhanced brain entropy as well as enhanced effective connectivity of the bottom-up informational flows [74].

## 6. Empirical findings

### (a) Psychedelic-induced brain state

Over the last decade, several neuroimaging studies have been carried out exploring the neural correlates of the psychedelic experience across various substances. To this date, studies have investigated brain activity under the influence of psilocybin—in both healthy [75] and depressed populations [76,77], LSD [78], ayahuasca [79] and DMT [80]. These data provide a unique opportunity to investigate the changes in spontaneous brain activity during the psychedelic experience across space and time.

**Figure 4.**
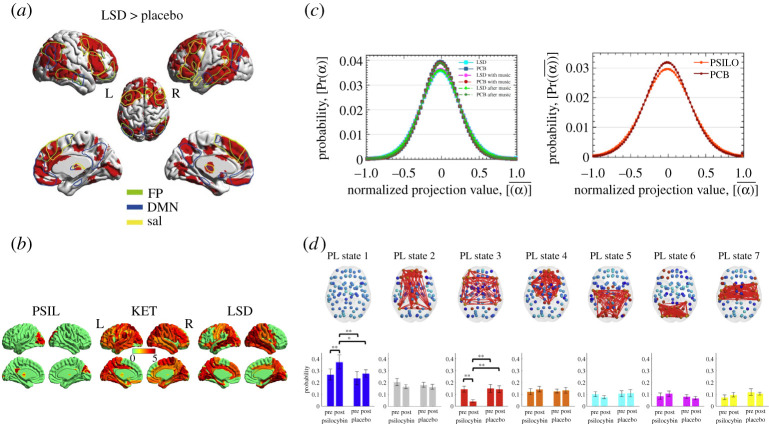
Psychedelic-induced state in space and time. Neuroimaging studies demonstrating various aspects of spatio-temporal dynamics under the influence of psychedelics. (*a*) LSD increases dynamic functional density, defined by averaged static functional connectivity between a region of interest and the rest of the brain, specifically in functional systems pertaining to the frontoparietal, default mode and salience networks [81]). (*b*) Repertoire broadening of brain substates, as described by connectome harmonics, in LSD and psilocybin-induced states [49]). (*c*) Temporal complexity, as defined by LZ-complexity, increases under psilocybin (PSIL), ketamine (KET) and LSD-induced states [82]. (*d*) Spatio-temporal dynamics alterations, as described by LEiDA, under the influence of psilocybin. Frontoparietal network becomes less frequently visited [83]. (Online version in colour.)

Along the spatial dimension, FC of fMRI activity has been demonstrated to broaden the repertoire of possible brain patterns, as described by connectome harmonics, both in the LSD and psilocybin states [46,49] (figure 4*c*). Similarly, an enhanced repertoire of dynamic connectivity substates has been observed under the influence of psilocybin [84]. Furthermore, another study on the effects of psilocybin using algebraic topology has demonstrated an increase in the number of low stability homological structures as well as an emergence of unique and stable homological structures [85].

From the perspective of functional systems, psilocybin has been found to decrease FC between the medial prefrontal cortex (mPFC) and posterior cingulate cortex, as well as functional activity of anterior cingulate cortex and mPFC [75]. Further, increases in between-network connectivity have been observed in most of the RSNs with the exception of lower cognitive networks [86]. Similarly, LSD increased FC density in higher associative networks matching with the DMN, salience and frontoparietal attention networks and thalamus, as well as between-network connectivity of the aforementioned networks and their lower cognitive counterparts [81] (figure 4*a*). This has been complemented by decreases in within-network connectivity of the DMN and other RSNs [78]. Using measures from graph-theory, LSD has been found to increase global integration [84], while ayahuasca has been found to increase the Shannon entropy of the degree distribution [87]. Taken together, these results point to within-network disintegration coupled with increased between-network cohesion.

Along the temporal dimension, signal complexity has been demonstrated to increase in LSD, psilocybin and ketamine-induced states [82] (figure 4*b*), while in the LSD state, this increase has been pronounced the most in the eyes closed condition [88]. Moreover, changes in temporal correlations have been observed in co-activations of various brain substates as described by connectome harmonic decomposition, suggesting a spatial grouping in a non-trivial manner [46]. Interestingly, EEG experiments have revealed that ayahuasca decreases collective oscillations in the alpha frequency band (8–13 Hz) and increases localized gamma power (30–100 Hz) [89]. Similarly, DMT was found to decrease both alpha and beta (13–30 Hz) band oscillations and increase signal diversity [80]. Lastly, in both LSD- and psilocybin-induced states, MEG signal power was decreased across the whole frequency spectrum [78,90]. Since the oscillations detected with EEG/MEG are generated by the synchronized activity of large neuronal populations, these studies suggest that the psychedelic experience is linked to an inhibition of long-range synchronization, leading to increased signal diversity, which in turn results in a broader repertoire of brain substates.

### (b) Depressive state

Recent developments in non-invasive neuroimaging have started to paint a system-level perspective of brain function in different brain disorders [91]. In major depressive disorders (MDD), aberrant functional network interactions have been associated with the control network responsible for cognitive control and outward interaction with the world, the DMN engaged in internal mental processes and introspection and the salience network involved in evaluating valence of relevant cognitive and biological events. Indeed, a description of the interactions among these three functional networks, dubbed the triple-network model, has been proposed to explain affective and cognitive dysfunction in several major brain disorders [92].

**Figure 5.**
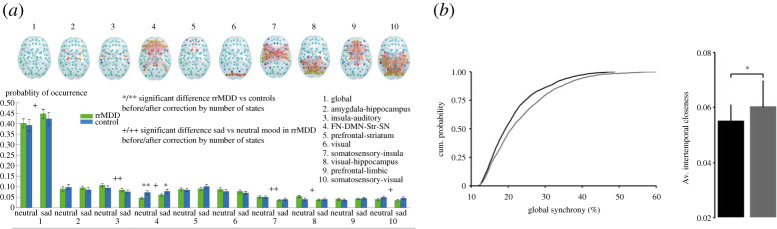
Depressive state in space and time. Neuroimaging studies demonstrating various aspects of spatio-temporal dynamics in major depressive disorder. (*a*) Spatio-temporal dynamics alterations, as described by leading eigenvector dynamics analysis. A brain network consisting of frontoparietal, default-mode salience and striatum regions becomes visited less frequently and for shorter periods of time while the globally active network is more prevalent in vulnerable remitted-MDD patients compared to healthy controls [93]. (*b*) Global synchrony and temporal stability are both increased in MDD patients [94]. (Online version in colour.)

A recent study has shown that vulnerable remitted-MDD patients were found to exhibit impaired recruitment and duration of a network consisting of frontoparietal, default-mode, salience and striatum regions, while concomitantly spending more time in a globally active network pattern compared to controls (figure 5*a*). The former network has been considered important for switching between internally and externally oriented attention [93]. Notably, when both patients and controls were induced in a sad mood by recalling sad past events, both groups exhibited an increased occupancy of the globally synchronized pattern, suggesting that mood modulates functional network dynamics. These results are in line with reports of an increased within-network connectivity in DMN regions, while more persistent resting-state FC between prefrontal and temporal regions of the DMN indicates a stronger prevalence of the DMN [94,95] (figure 5*b*). In terms of switching capabilities at the system level, an increase in variability has been observed between mPFC of the DMN and anterior insula and decreased variability between DMN and FPN suggestive of enhanced sensitivity to emotional information resulting in a ruminative state [94,95]. Overall, more pronounced synchronization and temporal stability has been observed in MDD patients compared to healthy participants, but further studies will be required to further investigate FCD in the depressive state.

### (c) Future perspective

Building on the insights from neuroimaging studies and whole-brain models, the brain's spatio-temporal dynamics can be perceived as a temporal trajectory through an n-dimensional dynamic landscape of weakly coupled substates constrained by the structural connectome. Furthermore, it happens at the edge of instability where the brain can explore a plethora of substates and maintain long-range temporal correlations. The characteristics of individual basins of attraction (substates) are described in terms of their prominence of occurrence (fractional occupancy), temporal stability (dwell times) and proximity to other substates (transition probability). Corroborated by neuroimaging studies in healthy brain functioning, the landscape will manifest enough stability to meaningfully visit substates, but at the same time sufficient flexibility not to become trapped in one particular substate. In the depressive state, alterations in the dynamical landscape will change the temporal trajectories with certain attractors being more or less prominent implying aberrant dwellings in certain parts of the landscape. On the contrary, the psychedelic state will result in the ‘flattening’ of the landscape with less predictable temporal trajectories implying novel re-routings through the underlying landscape [49,96–98].

**Figure 6.**
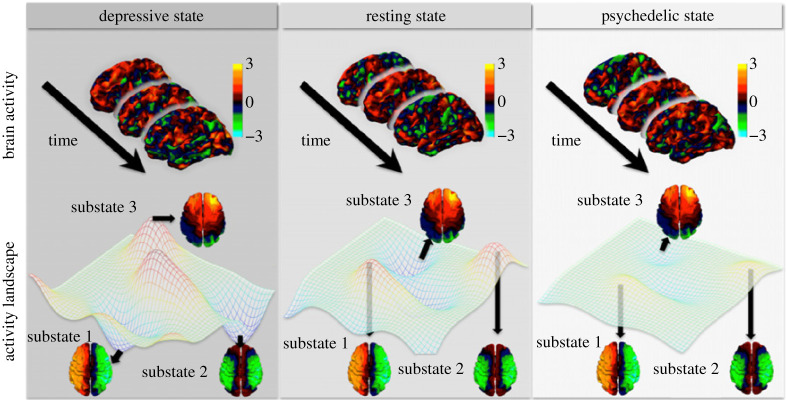
Activity landscape. Brain activity in different brain states as described by fMRI. Here, the depressive state, resting-state and psychedelic state. Activity landscape where the brain's spatio-temporal dynamics can be perceived as a temporal trajectory through an n-dimensional terrain of weakly coupled substates constrained by the structural connectome. Optimal healthy functioning is expected to be observed in the resting-state with enough stability and flexibility. In depressive states, specific attractors become pronounced, making it more difficult to escape from their vicinity. On the contrary, psychedelic-induced states will result in a ‘flattened’ landscape and thus will allow for more flexibility to move within the landscape (adapted from [49]). (Online version in colour.)

In practice, further progress will require mechanistic scenarios where various brain states can be modelled to their spatio-temporal description. This can be made possible endowing causal whole brain models with additional metadata reflecting heterogeneous features of brain organization such as neurotransmitter densities, excitatory/inhibitory ratio and temporal processing hierarchy [99]. Already promising studies have shown a causal link between 5HT-2A receptors transmission and the psychedelic-induced state in a whole-brain model paradigm [100,101]. Another important aspect will require causal understanding of how different brain states can transitions between each other both in terms of their spatio-temporal signatures, on the level of functional systems and neurotransmitter neurophysiology. For example, recent work has demonstrated how whole-brain causal models can be used in predicting regional significance in transiting between ordinary awake and dreamless sleep states [70]. Lastly, a further detailed theoretical description of brain states in terms of their functional hierarchies as well as their spatial and temporal multiscale representation will be relevant in constraining the space of mechanistic perturbation sites through which transitions between states are explored. For example, recent work demonstrating the brain's hierarchical nature has been developed in terms of functional harmonics—a method describing FC in terms of multidimensional and multiscale modes [102] (figure 6).

## 7. Conclusion

In this review, we have argued for a perspective of the brain as a complex system, reinforcing a clear need to interpret and understand the underlying mechanisms of brain states along both spatial and temporal dimensions. Importantly, this is made possible with non-invasive imaging and whole-brain modelling, that map and simulate the rich spatiotemporal dynamics of the brain. Experimentally, the optimal waking state is hypothesized to have a sustained stability and at the same time is conducive to flexible reorganizations. In this context, psychedelics-induced state and the depressive state will lie on the opposite sides of a spectrum of spatio-temporal dynamics.

## Data Availability

This article has no additional data.
